# Binarized Neural Network with Silicon Nanosheet Synaptic Transistors for Supervised Pattern Classification

**DOI:** 10.1038/s41598-019-48048-w

**Published:** 2019-08-12

**Authors:** Sungho Kim, Bongsik Choi, Jinsu Yoon, Yongwoo Lee, Hee-Dong Kim, Min-Ho Kang, Sung-Jin Choi

**Affiliations:** 1 Department of Electrical Engineering, Sejong University, Seoul, 05006 Korea; 2 School of Electrical Engineering, Kookmin University, Seoul, 02707 Korea; 3 Department of Nano-process, National Nanofab Center (NNFC), Daejeon, 34141 Korea

**Keywords:** Electrical and electronic engineering, Electronic devices

## Abstract

In the biological neural network, the learning process is achieved through massively parallel synaptic connections between neurons that can be adjusted in an analog manner. Recent developments in emerging synaptic devices and their networks can emulate the functionality of a biological neural network, which will be the fundamental building block for a neuromorphic computing architecture. However, on-chip implementation of a large-scale artificial neural network is still very challenging due to unreliable analog weight modulation in current synaptic device technology. Here, we demonstrate a binarized neural network (BNN) based on a gate-all-around silicon nanosheet synaptic transistor, where reliable digital-type weight modulation can contribute to improve the sustainability of the entire network. BNN is applied to three proof-of-concept examples: (1) handwritten digit classification (MNIST dataset), (2) face image classification (Yale dataset), and (3) experimental 3 × 3 binary pattern classifications using an integrated synaptic transistor network (total 9 × 9 × 2   162 cells) through a supervised online training procedure. The results consolidate the feasibility of binarized neural networks and pave the way toward building a reliable and large-scale artificial neural network by using more advanced conventional digital device technologies.

## Introduction

Although relatively little is known about the principle of information processing in the brain, it is certain that the information flows from neuron to neuron through synapses which have adjustable connection strengths (*i*.*e*., synaptic weights). The learning process in the brain is consequently the reconfiguration of the synaptic weights in the neural network, where the weights are updated in an analog manner. Based on this fact, several learning rules regulating the evolution of the synaptic weights have been proposed (such as spike-timing-dependent plasticity^1^), and recently, intensive efforts have been made to implement an electronic synaptic device that can emulate the functionality of synapses. The final goal of this research, which has been named neuromorphic engineering, is the realization of innovative computing architecture (neuromorphic system) based on an artificial neural network to overcome the energy inefficiency of conventional von Neumann architecture, by mimicking both the functional and structural characteristics of the biological systems^2,3^.

To date, the most promising candidates for a synaptic device are two-terminal resistive switching devices, *i*.*e*., memristors^4^. With memristors, analog conductance states can be modulated by using only a minuscule amount of energy consumption and can be maintained over the long term, which indicates the promising feasibility of emulating biological synapses^5–9^. Furthermore, by applying such memristors, primitive levels of artificial neural networks (*i*.*e*., synaptic device arrays) have been demonstrated experimentally for the application of pattern classification^8^, analog-to-digital conversion^10^, principal component analysis^11^, sparse coding calculations^12^, reservoir computing^13^, *K*-means data clustering^14^, and differential equation solver^15^. However, the on-chip implementation of neuromorphic systems with emerging synaptic devices is still very challenging due to the instability of analog weight modulation in a synaptic device, which has been identified in recent simulation studies^16,17^: although the neuromorphic systems are capable of tolerating the device-to-device variation or noise to a certain degree^18–20^, intrinsic nonlinearity and uncontrollability of analog conductance switching behavior critically degrades the performance of the system^16,17,20,21^. Unfortunately, this issue is common to almost all memristors and could not be solved by further optimizing the fabrication process or materials because the physical mechanism of the analog conductance modulation is typically an atomic-level random process based on electro/thermodynamics^22–24^. Although several methods for precise adjustment of the analog weight have been proposed^25–27^, these methods require a specially designed pulse waveform and impractical complex peripheral circuitry. In addition, recent memristors exhibit improved reliability^28–30^, but the fabrication process of the device is complex or the materials used are incompatible with conventional silicon processes, is a critical obstacle to the design of peripheral circuits.

Alternatively, the sustainability and reliability of digitally switching devices have been guaranteed over the past 20 years^31^. For example, in the case of the present NAND flash technology, stable multiple memory states with 3-dimensional stackability have already been applied to a product. Particularly, the density of the NAND flash already exceeds 2 × 10^9^ bits/mm^2^ ^32^, close to the density of synapses in the human frontal cortex (1.1 × 10^9^ synapses/mm^3^)^33^. Therefore, if the well-qualified conventional digital devices can contribute to a synaptic device, the goal of achieving on-chip implementation of a neuromorphic system can be realized sooner. Here, we demonstrate a binarized neural network (BNN) where the synaptic device is a more advanced digital-type switchable device, that is, a gate-all-around (GAA) silicon nanosheet transistor. A developed training/recognition algorithm of BNN enables the task of pattern classification with a supervised online training scheme. In this study, BNN is applied to three proof-of-concept examples: (1) handwritten digit classification (MNIST dataset^34^) verified by the simulation, (2) face image classification (Yale dataset^35^) verified by the simulation, and (3) 3 × 3 binary pattern classifications by using an integrated two 9 × 9 synaptic transistor arrays. The simulation and experimental results consolidate the feasibility of BNN and pave the way toward building a reliable, large-scale, and practical neuromorphic system from advanced conventional digital device technologies.

## Results and Discussion

**Figure 1:**
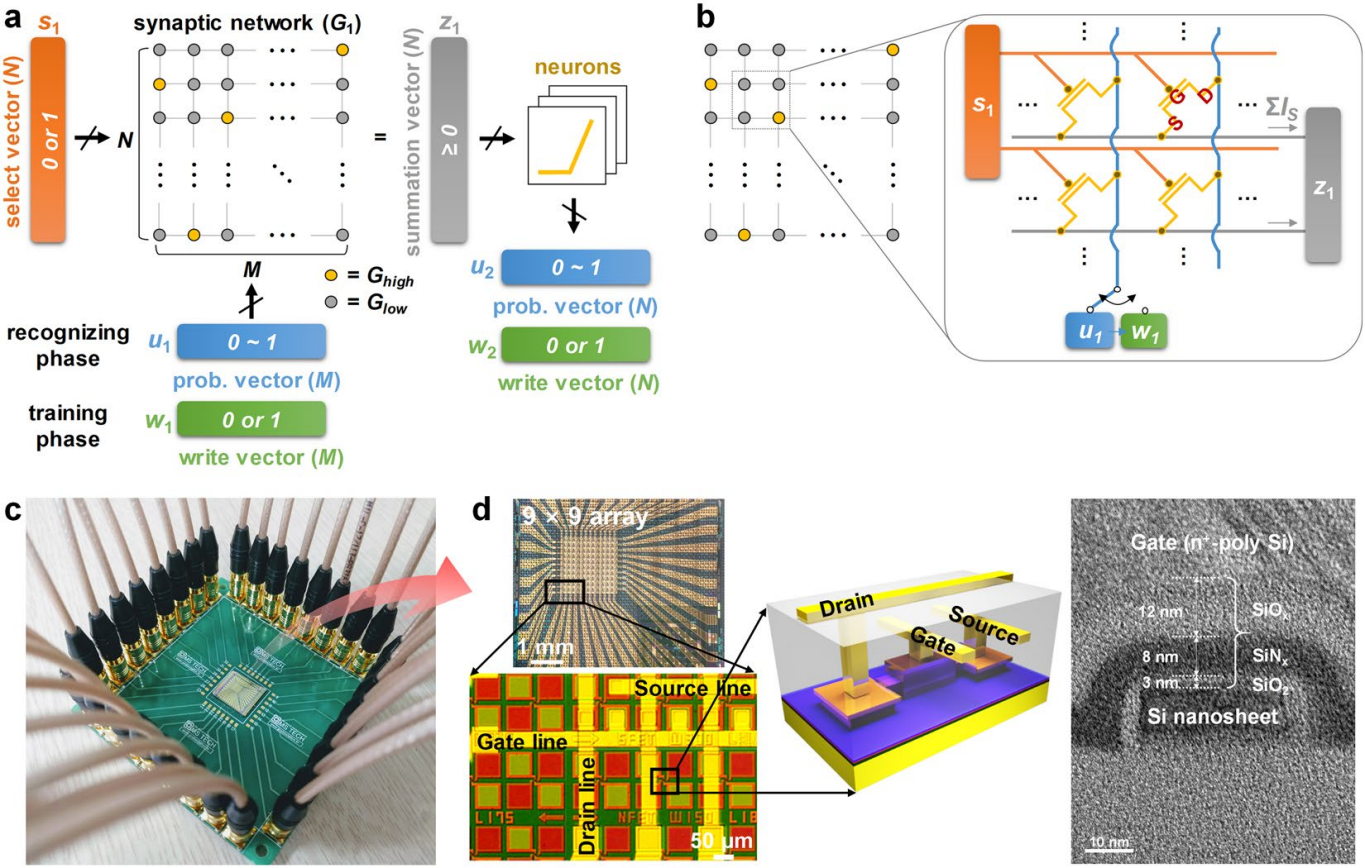
(**a**) The architecture of the binary neural network with *M* inputs and *N* outputs. The input pattern information corresponds to the *u*_1_(*i*) and *w*_1_(*i*), the *s*_1_(*i*) enables supervised training by selecting a specific row, and the *z*_1_(*i*) is the output of the network. (**b**) The schematic of synaptic transistor array, where *s*_1_(*i*) involves *V*_*G*_, and either *u*_1_(*i*) or *w*_1_(*i*) involves *V*_*D*_. Integrated *I*_*S*_ in a row direction corresponds to *z*_1_(*i*). (**c**) The photo of the test board with an integrated synaptic transistor array. (**d**) The optical and transmission electron microscope images of the synaptic transistor. The SiN charge trap layer embedded in the gate dielectric enables the digital-type channel conductance switching with high reliability.

Figure 1a depicts the architecture of BNN^36^ with *M* inputs and *N* outputs. Synaptic weights in the network *G*_1_(*i*, *j*) are given within one binary value: *G*_1_(*i*, *j*)  l{*G*_*high*_ or *G*_*low*_}; *G*_*high*_ and *G*_*low*_ represent the high- and low-conductance states of the synaptic device, respectively (subscripted numbers indicate the order of each network when multiple networks are involved). The input pattern information is delivered into the network by two types of vectors: *u*_1_(*i*) and *w*_1_(*i*) denote the probability- and write-vector, respectively. When an input pattern needs to be distinguished from previously trained patterns (*i*.*e*., recognizing phase), *u*_1_(*i*) is applied to the network. *u*_1_(*i*) corresponds directly to each pixel of information of the input pattern such as the intensity, which is rescaled to 0 ≤ *u*_1_(*i*) ≤ 1. When an input pattern needs to be trained by updating the synaptic weight (*i*.*e*., training phase), *w*_1_(*i*) instead of *u*_1_(*i*) is applied to the network, where *w*_1_(*i*)  w{0 or 1} is stochastically determined by learning probability *p* le*γ*∙*u*_1_(*i*) (where *γ* is the learning rate, and *u*_1_(*i*) is used as a probability value to decide *w*_1_(*i*)). Here, the weight updating of BNN is conducted in a supervised manner. To this end, select-vector *s*_1_(*i*) le{1 or −1 or 0} directs the training of the input pattern according to its label, where *s*_1_(*i*) he1, −1, and 0 represent ‘potentiation’, ‘depression’, ‘no update’ of the synaptic weight, respectively. Finally, the resultant outcome of the network is the summation vector *z*_1_(*i*) given as ${\sum }_{j=1}^{M}{G}_{1}(i,j){u}_{1}(i,j)$, which is the sum of the products of *G*_1_(*i*, *j*) and *u*_1_(*i*) in a row direction. The subsequent *u*_2_(*i*) and *w*_2_(*i*) of the next network are determined by passing *z*_1_(*i*) through the designed neuron function (the detail of the neuron is discussed later).

For the physical implementation of BNN, the GAA silicon nanosheet transistor contributes to a synaptic device, where the embedded charge trap layer (silicon nitride) in the gate dielectric enables adjustable digital-type channel conductance (*i*.*e*., synaptic weight modulation). The fabrication process, the device variability, and the digital-type switching performance are discussed in Supplementary Information Note 1. In the configuration of the synaptic transistor array (Fig. 1b), *s*_1_(*i*) corresponds to the gate voltage (*V*_*G*_) of the synaptic transistors in a particular row, and either *u*_1_(*i*) or *w*_1_(*i*) corresponds to the drain voltage (*V*_*D*_). The source current of each synaptic transistor (*I*_*S*_) is determined by the channel conductance (*G*_*high*_ or *G*_*low*_) and *V*_*D*_, and consequently, the integrated *I*_*S*_ of each row ($\sum {I}_{S}=\sum G\cdot {V}_{D}$) represents *z*_1_(*i*). Figure 1c shows the implemented test board with an integrated synaptic transistor array, and Fig. 1d shows the microscope images of the synaptic transistors (the array measurement setup using a test board is presented in Supplementary Information Note 2).

**Figure 2:**
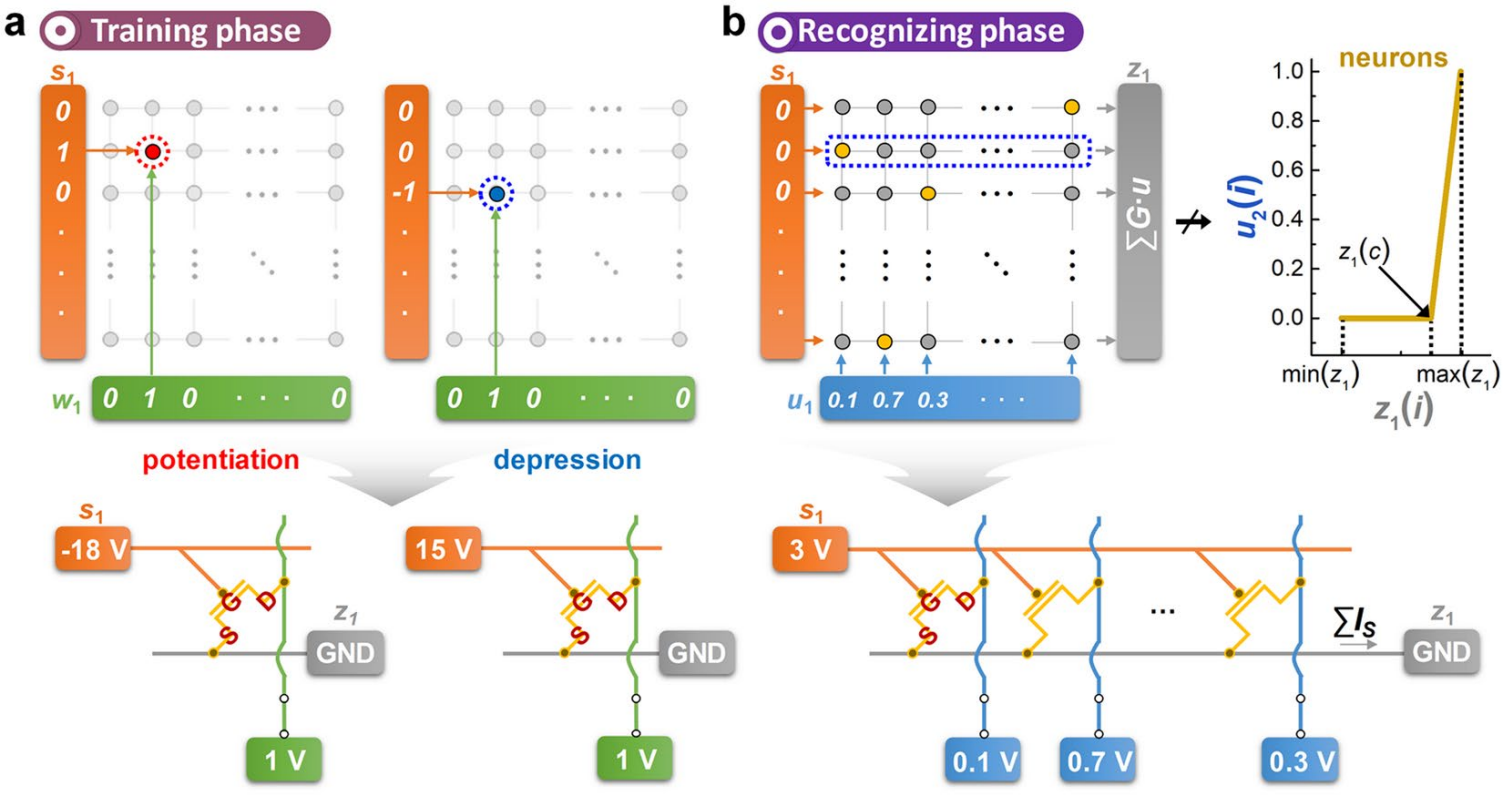
(**a**) The training phase of BNN: *G*_1_(*i*, *j*) is updated to *G*_*high*_ when *w*_1_(*i*)∙*s*_1_(*i*) wh1, updated to *G*_*low*_ when *w*_1_(*i*)∙*s*_1_(*i*) wh-1, and maintained its state when *w*_1_(*i*) · *s*_1_(*i*) wi0. Each element of *s*_1_(*i*) and *w*_1_(*i*) corresponds to *V*_*G*_ and *V*_*D*_, respectively. (**b**) The recognition phase of BNN: *u*_1_(*i*) represents *V*_*D*_, and *z*_1_(*i*) is utilized for either classifying the input pattern or determining *u*_2_(*i*).

BNN has two different modes of operation, *i*.*e*., training and recognizing phases. The training phase of BNN to update the synaptic weight (Fig. 2a) is conducted through the cooperation of *w*_1_(*i*) and *s*_1_(*i*), which leads to three different consequences: *G*_1_(*i*, *j*) is updated to *G*_*high*_ when *w*_1_(*i*)∙*s*_1_(*i*) wh1 (*i*.*e*., *w*_1_(*i*)  e1 and *s*_1_(*i*)  a1), updated to *G*_*low*_ when *w*_1_(*i*)∙*s*_1_(*i*) wh-1 (*i*.*e*., *w*_1_(*i*)  e1 and *s*_1_(*i*)  a-1), and maintains its state when *w*_1_(*i*)∙*s*_1_(*i*) ws0 (*i*.*e*., *w*_1_(*i*) .e0 or *s*_1_(*i*)  o0); these are referred to as ‘potentiation’, ‘depression’, and ‘no update,’ respectively. Because the higher learning probability *p* ( (c*γ*∙*u*_1_(*i*)) leads to *w*_1_(*i*) becoming 1 more often, the larger *u*_1_(*i*) results the potentiation/depression of synaptic weight more frequently. In terms of synaptic transistor operation, *s*_1_(*i*) n {1, −1, 0} corresponds to *V*_*G*_   −18 V, 15 V, and 3 V, respectively. Similarly, *w*_1_(*i*)  S{0, 1} corresponds to *V*_*D*_ 0,floating and 1 V, respectively. Consequently, *w*_1_(*i*)∙*s*_1_(*i*) o {1, −1, 0} leads to ‘increase’, ‘decrease’ and ‘maintain’ the channel conductance of the synaptic transistor, respectively, according to the configuration of *V*_*G*_ and *V*_*D*_.

Next, the recognizing phase is conducted by applying *u*_1_(*i*) to the network instead of *w*_1_(*i*), as shown in Fig. 2b (since the weight update is not required during the recognizing phase, all *s*_1_(*i*) are set to 0). The purpose of the recognizing phase is twofold: (1) classification of the input pattern by matching with previously trained patterns, and (2) generation of *u*_2_(*i*) for transferring the input pattern information to the next network. As mentioned above, *u*_1_(*i*) involves each pixel of information of the input pattern, and the resultant *z*_1_(*i*) is the sum of *G*_1_(*i*, *j*)∙*u*_1_(*i*) in a row direction. If *z*_1_(*i*) is the output of the last network, *z*_1_(*i*) is used to classify the input pattern. The maximum *z*_1_(*i*) indicates the estimated label for a given input pattern (the detail classification process will be discussed in later). However, when multiple networks are involved in the system, *u*_2_(*i*) of the next network is generated by exploiting *z*_1_(*i*). In detail, *u*_2_(*i*) is determined by passing *z*_1_(*i*) through the designed neuron function: *u*_2_(*i*) is zero when *z*_1_(*i*)  i*z*_1_(*c*), and *u*_2_(*i*) is increased linearly to 1 when *z*_1_(*i*) ≥ *z*_1_(*c*). A critical point, *z*_1_(*c*), is given according to the total number of labels (*l*) (*e*.*g*., *l*  g10 in MNIST dataset, and *c* nd*N*/*l*). Because of the discontinuity of the neuron function, a relatively small value of *z*_1_(*i*) cannot be delivered to the next network. In other words, only meaningful information (features) of the input pattern can be transferred to the next network, which increases the classification accuracy by introducing multiple (deeper) networks. In terms of synaptic transistor operation, *u*_1_(*i*) corresponds directly to *V*_*D*_ ranged from 0 to 1 V. Then, integrated *I*_*S*_ row by row represents *z*_1_(*i*).

**Figure 3:**
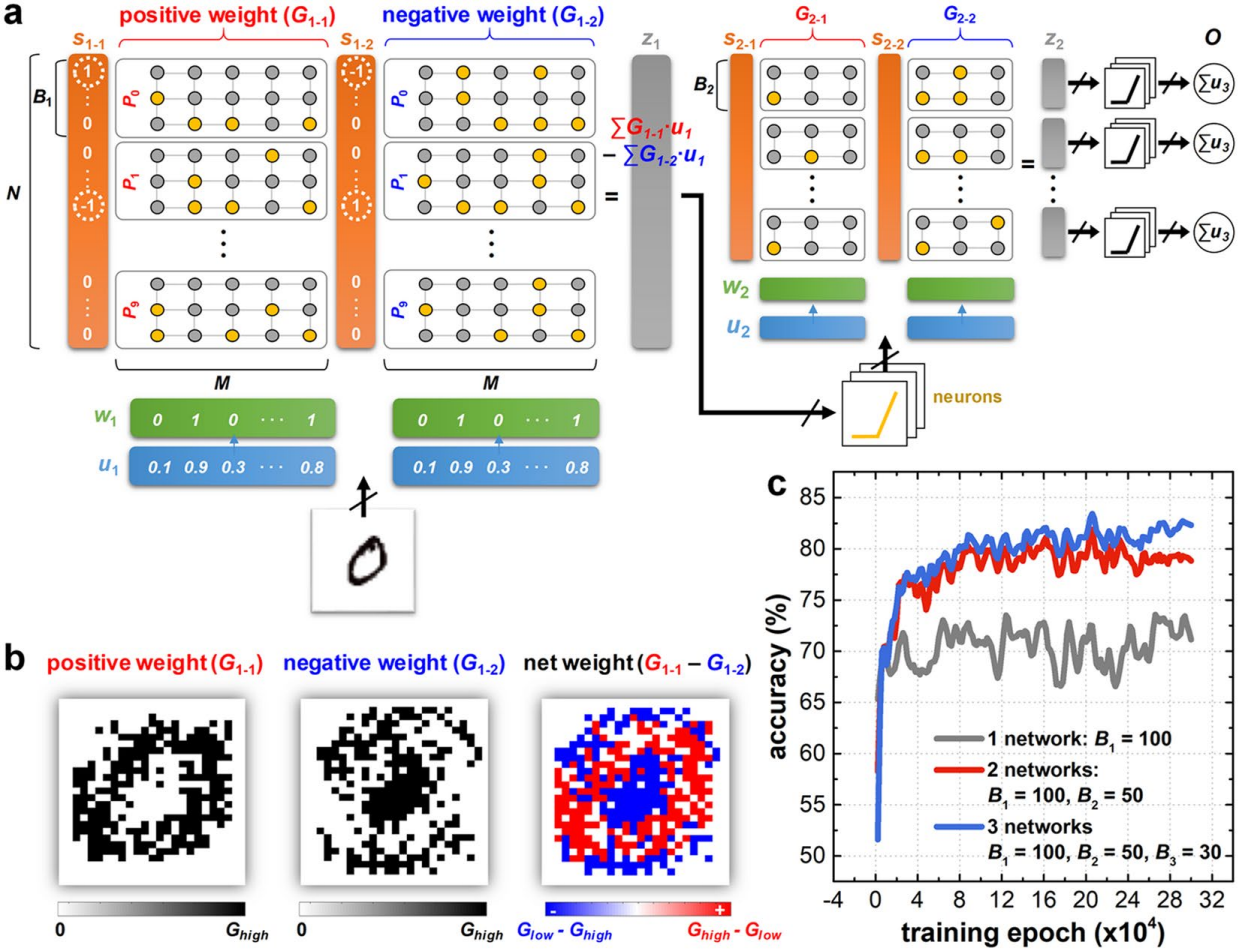
(**a**) Schematic of the network architecture for handwritten digit classification with two networks (*G*_1_ and *G*_2_). Each network is divided into two subnetworks (*e*.*g*., *G*_1-1_ and *G*_1-2_) to represent positive and negative synaptic weights, respectively. This subnetwork is partitioned again to the buckets (*P*_0_ ~ *P*_9_), where each bucket is trained on the input patterns according to the label. (**b**) One example of synaptic weights after 60000 times of the training epoch: one row at the bucket *P*_0_ is selected from *G*_1-1_ and *G*_1-2_, and the resultant *G*_1-1_–*G*_1-2_ are plotted, respectively. (**c**) The evolution of classification accuracy as a function of the training epoch, which is also affected by the network configuration (*i*.*e*., number of networks, bucket size, learning rate). The learning rate *γ* of all results is 0.2.

In the following, the pattern classification ability of BNN is verified by three proof-of-concept examples: the first example is handwritten digit classification (MNIST dataset) verified by the simulation. Figure 3a shows the schematic of BNN including two networks (*G*_1_ and *G*_2_): note that the first network *G*_1_ is divided into two subnetworks, one of which represents a positive weight value (*G*_1-1_) and the other that represents a negative weight value (*G*_1-2_). Again, *G*_1-1_ and *G*_1-2_ are partitioned into buckets (depicted as *P*_0_ ~ *P*_9_, the size of each bucket is *B*_1_). Each bucket is assigned to train only a specific input pattern according to the label (*e*.*g*., digit ‘0’ pattern is only trained at the bucket *P*_0_). Because the total labels (*l*) of the MNIST dataset are 10, *G*_1_ is accordingly partitioned into 20 buckets and *N* ar*l*∙*B*_1_. Under this configuration, each pixel intensity value of the MNIST dataset (28 × 28 pixels) is rescaled to the range between 0 and 1, which becomes *u*_1_(*i*) as it is (*i* (a1 to *M*, *M*  t784). Then, *w*_1_(*i*) is given by *u*_1_(*i*) according to the learning probability *p*. Next, to generate *s*_1-1_(*i*) and *s*_1-2_(*i*) for adjusting the weights properly, the following steps are conducted sequentially (Fig. 3a). Step 1: in *G*_1-1,_ one row (*r*_1_^th^ row) is randomly selected from the bucket belonging to the label of the input pattern, and *s*_1-1_(*r*_1_) is set to 1. Step 2: in *G*_1-1_, another row (*r*_2_^th^ row) is randomly selected from the buckets that do not belong to the label of the input pattern, and *s*_1-1_(*r*_2_) is set to −1. Step 3: all *s*_1-1_(*i*) except *i*  e*r*_1_ and *r*_2_ are set to 0. Step 4: *s*_1-2_(*i*) of *G*_1-2_ is given as −*s*_1-1_(*i*). Following these sequences, a chosen input pattern is trained only in the *r*_1_^th^ row of *G*_1-1_ during Step 1. However, since the weight of *r*_1_^th^ row is only potentiated due to *s*_1-1_(*r*_1_) = −1, most of the weight will be potentiated if the training phase is repeated continuously. Therefore, during Step 2, the weight of *r*_2_^th^ row of *G*_1-1_ should be depressed according to the input pattern. Interestingly, because *s*_1-2_(*i*) = −*s*_1-1_(*i*), the bucket of *G*_1-2_ is trained oppositely to the bucket of *G*_1-1_ during Step 3 and Step 4. For example, digit ‘0’ pattern is trained at the bucket *P*_0_ in *G*_1-1_. In contrast, symmetrical *P*_0_ in *G*_1-2_ is trained to the features of other digits (*e*.*g*., ‘1’ to ‘9’). Consequently, the resultant *z*_1_(*i*), defined as ${\sum }_{j=1}^{M}{G}_{1-1}(i,j){u}_{1}(i,j)-\,{G}_{1-2}(i,j){u}_{1}(i,j)$, contains the feature information of the input pattern corresponding to the label excluding the features other than itself.

The training phase of the second network *G*_2_ is the same as the training phase of *G*_1_. The only difference is, if *G*_2_ is the last network, *z*_2_(*i*) results in the final output *O*(*i*) are given by the sum of the neuronal output over the rows from the bucket of each label. The maximum *O*(*i*) designates the estimated label for a given input pattern. Accordingly, the classification accuracy is evaluated regarding agreement between the desired and estimated labels. Figure 3b shows one example of synaptic weights after the training of the MNIST dataset is finished, *i*.*e*., one arbitrarily selected row at the bucket *P*_0_ in *G*_1-1_ and *G*_1-2_. The synaptic weights of *G*_1-1_ contain the feature of digit ‘0’ pattern. In contrast, the synaptic weights of *G*_1-2_ contain the features of other digits except ‘0’. The net synaptic weight (*G*_1-1_–*G*_1-2_) has both positive and negative values, which helps to improve the classification accuracy by emphasizing a distinctive feature of the digit ‘0’ pattern (the impact of negative synaptic weight *G*_1-2_ on the classification accuracy is discussed in Supplementary Information Note 3). Finally, the classification accuracy of the MNIST dataset is shown in Fig. 3c as a function of the training epoch, where the number of networks alters the accuracy. With a single network, the accuracy merely reaches approximately 70% with *B*_1_ it 100, while deploying one more network improves the accuracy up to approximately 80% with *B*_1_ it 100, *B*_2_  050. Improvement in the accuracy continues onwards with more networks (*e*.*g*., three networks; blue curve in Fig. 3c), although the effect decreases. Additional accuracy tests depending on different parameters (*e*.*g*., learning rate or bucket size) are presented in Supplementary Information Note 4.

**Figure 4:**
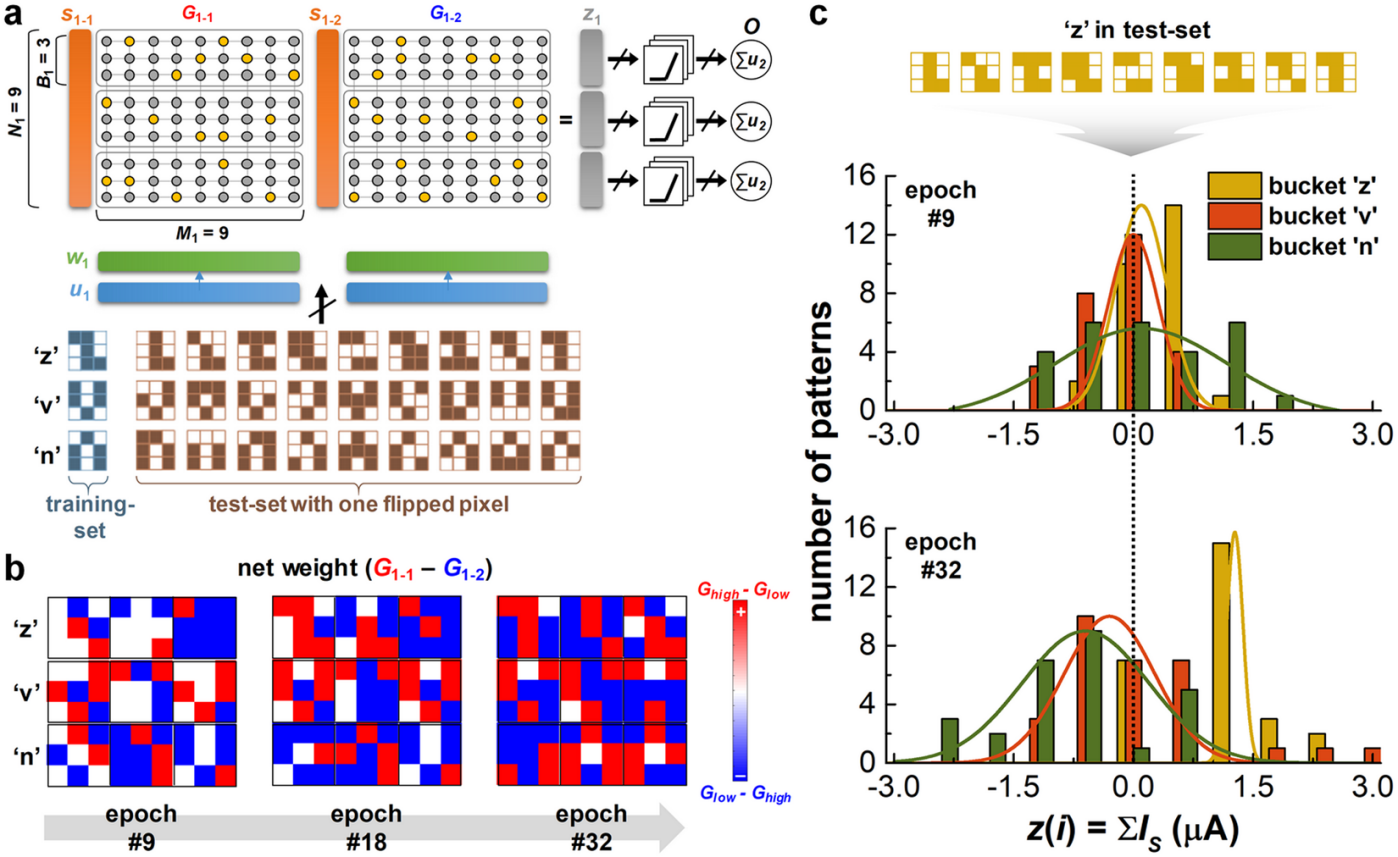
(**a**) Schematic of the network architecture for 3 × 3 binary pattern classification (the letters ‘z’, ‘v’, and ‘n’) with integrated 162 (9 × 9 × 2) synaptic transistors. The training set used in the training phase consisted of 3 correct patterns. The test set used in the recognizing phase (to evaluate the classification accuracy) consisted of 27 patterns with one flipped pixel from the training set. (**b**) The evolution of net synaptic weights (*G*_1-1_–*G*_1-2_) is a function of the training epoch. (**c**) Measured results of obtained *z*(*i*) (*i*.*e*., integrated *I*_*S*_ in row direction) when the training epoch is 9 and 32. When the test pattern ‘z’ is applied to the network during the recognition phase, the resultant *z*(*i*) is different from that of each bucket; the *z*(*i*) obtained from bucket ‘z’ is obviously larger than the others.

The second example is the face image (Yale dataset) classification. Because the classification procedure is exactly equal to that of the MNIST dataset discussed above, the results will be discussed in Supplementary Information Note 5. The last example is the experimental demonstration of BNN, where 3 different 3 × 3 binary patterns (denoted as the letters ‘z’, ‘v’, ‘n’)^8^ are classified. As shown in Fig. 4a, bucket size *B*_1_ is set to 3 (due to the limit of the fabricated array size), and thus *M*_1_ rr 3 × 3, *N*_1_ , 3 · *B*_1_, the total number of used synaptic transistors is 9 × 9 × 2 + 162 cells. By applying the supervised online training scheme discussed above, Fig. 4b shows the evolution of the weights as a function of training epoch. When the patterns in the training set, *i*.*e*., the patterns ‘z’, ‘n’, and ‘v’, are consecutively applied to the network during the training phase, each pattern is trained at the corresponding bucket of the network, which is defined as one training epoch. Then, to evaluate the pattern classification accuracy, the test set patterns (with one flipped pixel from the training set, the total number of patterns in the test set is 27) are applied to the network. Figure 4c shows resultant *z*(*i*) in a different training epoch (the data show only when the test pattern ‘z’ is applied to the network. The data for the test patterns ‘v’ and ‘n’ are presented in Supplementary Information Note 6). Note that the *z*(*i*) values obtained from each bucket are almost similar when the training epoch is only 9, which means that the test pattern ‘z’ cannot be classified properly. In contrast, after the training epoch is 32, *z*(*i*) obtained from bucket ‘z’ is much larger than the others, which indicates that the test pattern ‘z’ can be classified. When the training and recognizing phases are repeated, The classification accuracy is finally reached 100% after 24 times of the application of the training epoch (see Supplementary Information Note 6).

To classify the 3 different 3 × 3 binary patterns mentioned above, the number of synaptic transistors required in BNN (162 cells) is greater than the number of synaptic devices used in the previous memristor array^8^ (60 memristors). However, BNN is believed to be more appropriate for large-scale on-chip implementation due to the high controllability and sustainability of the digital-type conductance switching property, which has already been confirmed by the advanced conventional digital devices. In addition, because the synaptic transistor itself acts as a selector, the chronic problems in memristor crossbar arrays, such as a sneaky current path, can be solved without any further efforts. Moreover, a peripheral driving circuitry, as well as synaptic devices, can also be implemented using the equivalent device technology, which enables a considerably easier full-system integration.

In summary, the binarized neural network is implemented using a gate-all-around silicon nanosheet transistor that exhibits highly reliable and accurately controllable channel conductance modulation in a digital manner. With a supervised online training scheme, pattern classification tasks are experimentally demonstrated. Due to the use of advanced digital device technology, further monolithic integration with neuronal circuits and final brain-like cognitive computing system from an artificial neural network could be realized on a small chip. Considering only a single synaptic device, the demonstrated synaptic transistor in this study may require more energy consumption compared to existing memristors. However, considering the large-scale array of synaptic devices, the energy consumption from the sneaky-current flow will be more critical^37^. However, the existing memristors cannot prevent this problem completely without introducing an additional selector device. In contrast, transistor-based synaptic device arrays can avoid this issue without any further effort, which will certainly be beneficial in terms of system-level energy consumption. Therefore, the binarized neural network can provide the breakthrough for the device-level of the present neuromorphic system research based on analog-manner synaptic devices and enable us to provide a novel direction and inspiration for neuromorphic engineering in the future.

## Supplementary information

Supporting Information
