# Supplementary material for: Binarized Neural Network with Silicon Nanosheet Synaptic Transistors for Supervised Pattern Classification
Source: Sci Rep. 2019 Aug 12;9:11705. doi: 10.1038/s41598-019-48048-w (PMC6690903; doi:10.1038/s41598-019-48048-w)
Supplement: Supplementary file 1 — Supporting Information [file 41598_2019_48048_MOESM1_ESM.pdf]

Supplementary information for

Binarized Neural Network with Silicon Nanosheet Synaptic Transistors for Supervised Pattern Classification

Sungho Kim[1], Bongsik Choi[2], Jinsu Yoon[2], Yongwoo Lee[2], Hee-Dong Kim[1], Min-Ho Kang[3] and Sung-Jin Choi[2,*]

[1]Department of Electrical Engineering, Sejong University, Seoul 05006, Korea

[2]School of Electrical Engineering, Kookmin University, Seoul 02707, Korea

[3]Department of Nano-process, National Nanofab Center (NNFC), Daejeon 34141, Korea

*Correspondence to S. J. C. (sjchoiee@kookmin.ac.kr).

Note 1. Gate-all-around (GAA) silicon nanosheet synaptic transistor

1-1. Fabrication process

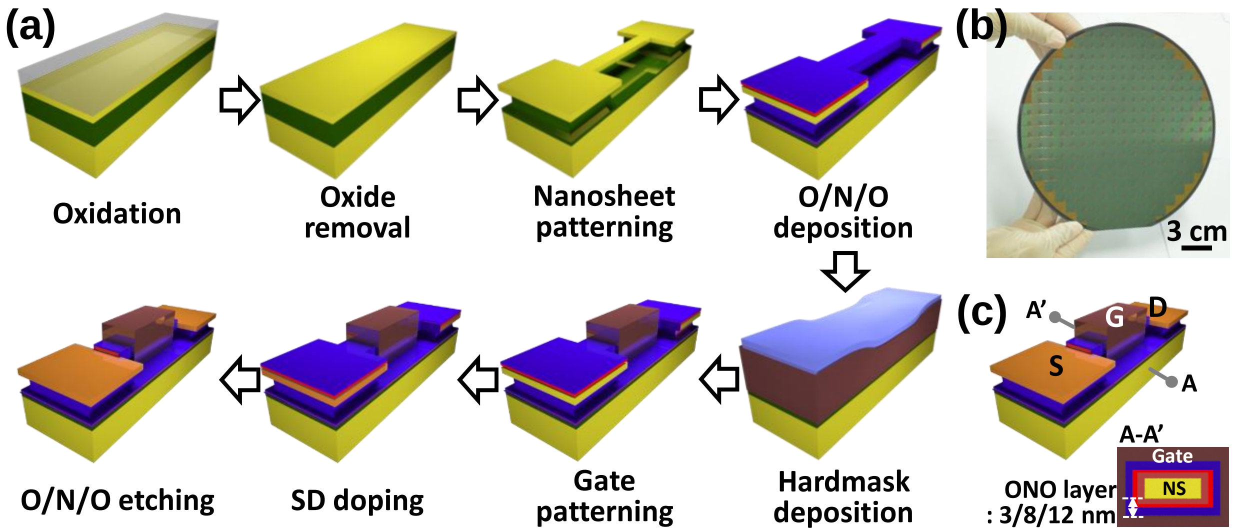

Figure S1. (a) The fabrication process of nanosheet synaptic transistor. (b) Fabricated synaptic transistors on 8-inch SOI wafer. (c) Schematic of the synaptic transistor and its cross-sectional view.

The fabrication process of a GAA nanosheet synaptic transistor on an 8-inch SOI wafer is shown in Fig. S1. The minimum thickness of the silicon nanosheet is approximately 5 nm, and the minimum gate length and nanosheet width are 39.6 nm and 26.4 nm, respectively. We embedded a silicon nitride (SiN) layer into the gate dielectric to enable precisely controllable channel conductance switching in a digital manner according to the charge storage in the SiN layer. The detail fabrication process is as follows: 1) silicon-on-insulator (SOI) wafer was used as a substrate. 2) By the oxidation process and subsequent etching process, the desired nanosheet (NSH) thickness can be controlled. Then, boron ions were implanted on the NSH to block the leakage path below the NSH channel, in a process known as channel stop implantation. 3) In order to form the NSH structure, iterative deep reactive-ion etching (RIE) was done under the optimum process conditions. 4) Oxide/nitride/oxide (ONO) layers were deposited using plasma

enhanced chemical vapor deposition (PECVD) as a charge storage layer. 5) Doped poly-Si for the gate was deposited using PECVD. Additionally, high-density plasma (HDP) oxide, which acts as a hard mask to etch poly-Si was deposited using PECVD. 6) Photolithography using a krypton fluoride (KrF) laser was conducted on a HDP oxide hard mask, after which the hard mask was etched to pattern the channel region. Then, a CMP process was conducted to etch the residual poly-Si, which results in the formation of the gate-all-around configuration. 7) Phosphorous ions were implanted to form the source/drain (S/D) electrodes. Self-aligned S/D implantation was carried out while the exposed gate blocked the dopants as the implant stopper. Thus excepting the region covered by the exposed gate, all other areas were heavily doped. 8) The remaining ONO layers were etched to open the S/D region. Importantly, the fabrication process undergoes few divergences compared to current FinFET technologies; hence, the GAA nanosheet synaptic transistors allow the realization of a high-density artificial neural network.

The reason for demonstrating a transistor with a nanosheet structure is as follows. FinFETs have successfully enabled continuous technology scaling from planar devices by improving gate to channel control at reduced gate length, resulting in an improved performance at low operating voltage. However, FinFETs are facing many challenges in patterning, device performance, layout, and cost for further scaling. By contrast, gate-all-around nanowire transistors (NW-FETs) are expected to further enable device scaling due to their better short-channel control as well as high current density. One possible limiting factor of NW-FETS is their lower drive currents from inherently smaller effective channel width. To solve this issue, nanosheet transistors (NSH-FETs) have been suggested recently to continue scaling. In NSH-FETs, the width of the silicon body is not limited by fin pitch and fin quantization, giving more freedom to achieve sufficient effective channel width. Therefore, Si nanosheet transistor was demonstrated in this study because the

performance of the nanosheet transistor is considered to be the best in the present technology. Of course, there is no particular advantage of a nanosheet structure in the operation of the binarized neural network. However, we believe that it is desirable to utilize the latest technology transistor when considering the compatibility with the peripheral circuits when fabricating the highly integrated neural network in the future.

1-2. Device variability

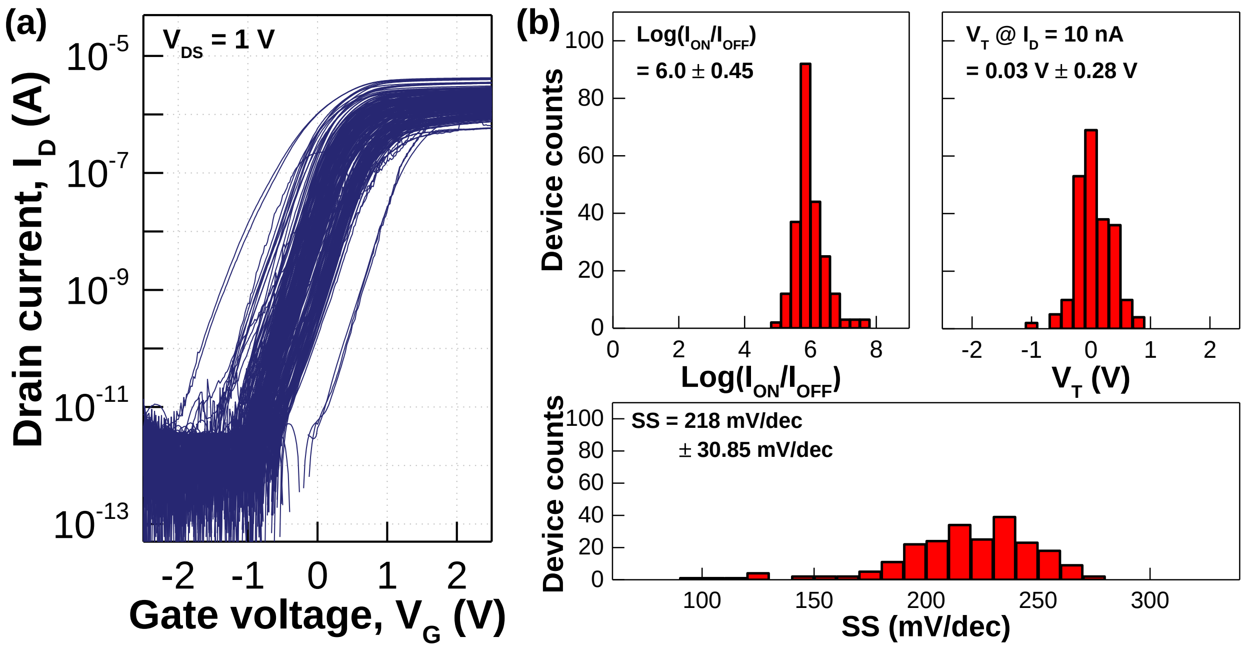

Figure S2. (a) Transfer characteristics of synaptic transistors measured from 242 cells. (b) Statistical performance data of synaptic transistor about I_{on}/I_{off}, V_T, and subthreshold swing (SS) respectively.

Fig. S2a shows the transfer characteristics of the GAA nanosheet synaptic transistors measured in the entire 8-inch wafer area, which exhibits good uniformity. In addition, Fig. S2b shows the statistical distribution of the synaptic transistor performances. Three parameters, I_{on}/I_{off}, V_T, and SS, have a good uniformity, which is superior to existing two-terminal memristors. This high uniformity with compatibility of conventional CMOS is essential for implementing a highly integrated on-chip level neuromorphic system.

1-3. Channel conductance switching behaviour

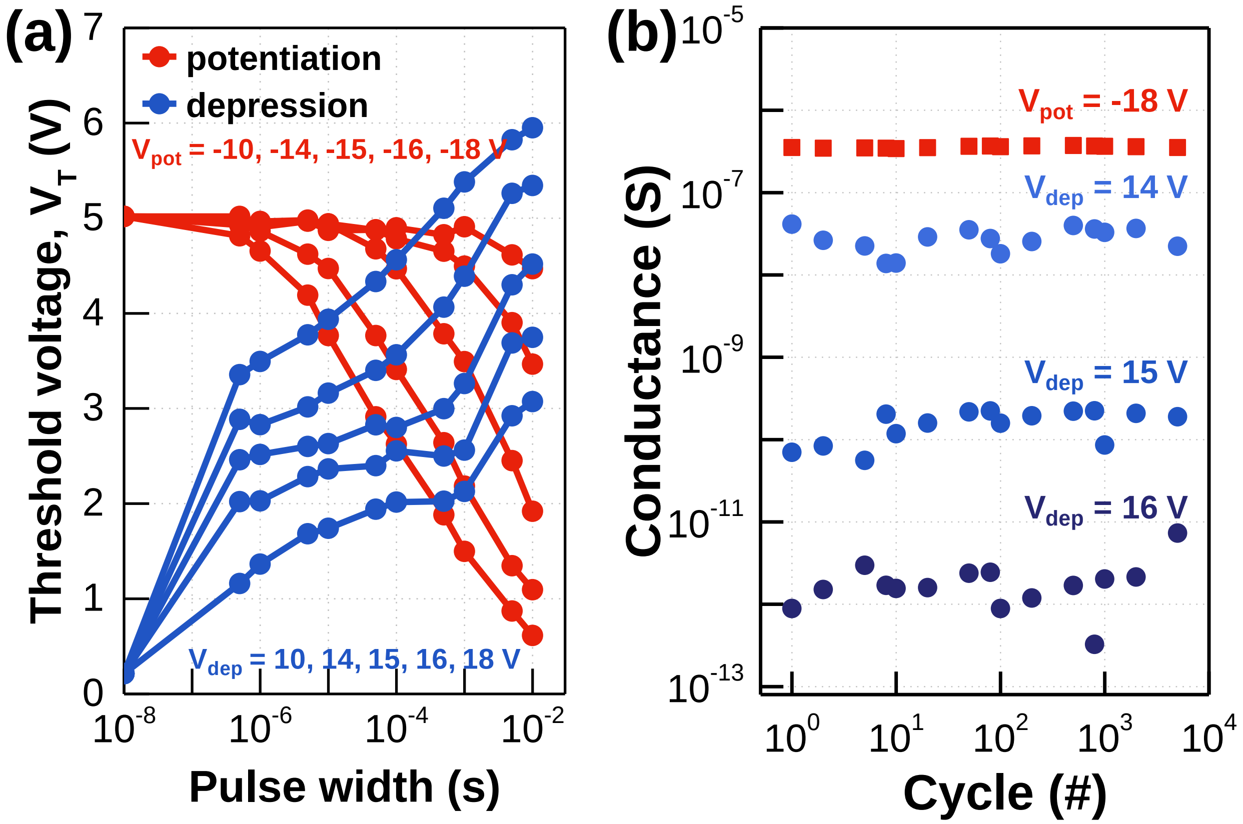

Figure S3. (a) Channel conductance switching behavior of synaptic transistor as a function of applied pulse width and level. (b) Switching cycle endurances of synaptic transistor with 100 μs pulse width.

The trapping/detrapping process of electrons in the SiN layer through the gate voltage (V_G) control results in channel conductance switching. Fig. S3a shows the evolution of V_T as a function of applied pulse width and level. Red curves denote the decrease of V_T, which indicates the increase of channel conductance (*i.e.*, potentiation). In contrast, blue curves denote the increase of V_T, which indicates the decrease of channel conductance (*i.e.*, depression). By adjusting the width and level of applied pulse, the channel conductance can be precisely controlled. Moreover, the difference in the conductance remains uniform even with thousands of switchings as shown in Fig. S3b, which confirms the high reliability and sustainability of digital-type weight modulation of demonstrated synaptic transistor.

1-4. Retention property of modulated channel conductance

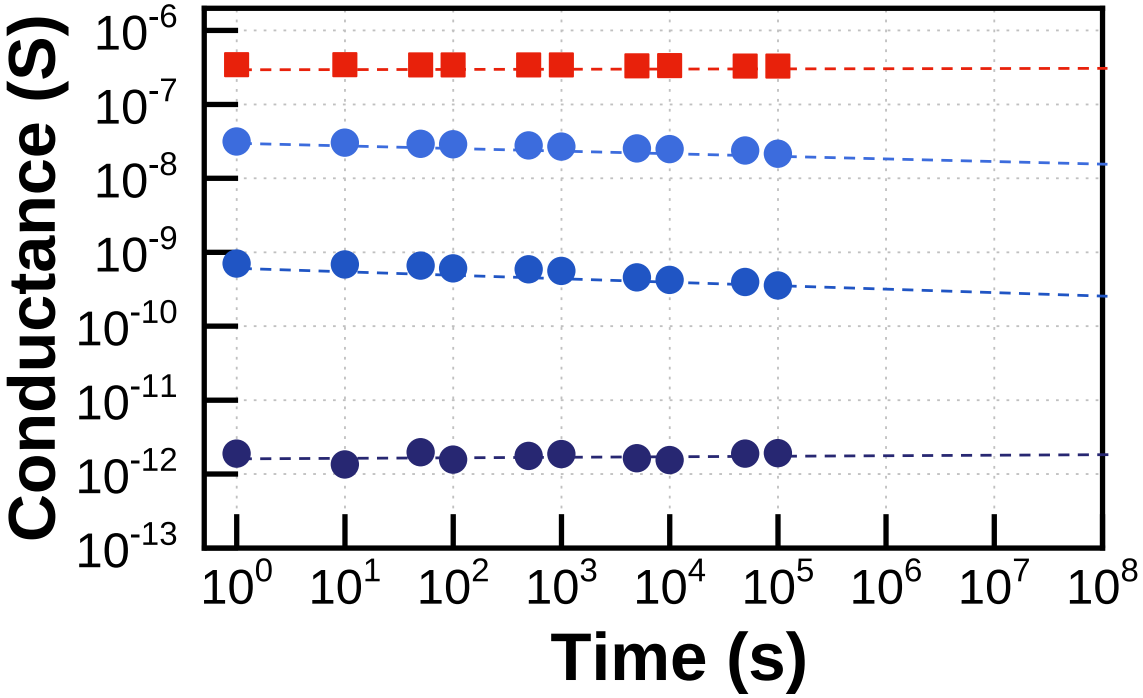

Figure S4. The retention property of Si nanosheet transistor measured at room temperature.

Fig. S4 shows the retention property of Si nanosheet transistor measured at room temperature. Note that the modulated channel conductance states show no sign of significant decay even 10^5 seconds after the application of the potentiation/depression pulses. This long-term behavior caused by the trapping/detrapping process of electrons in the SiN layer can guarantee the reliable operation of the proposed binarized neural network.

Note 2. The array measurement setup for the test board

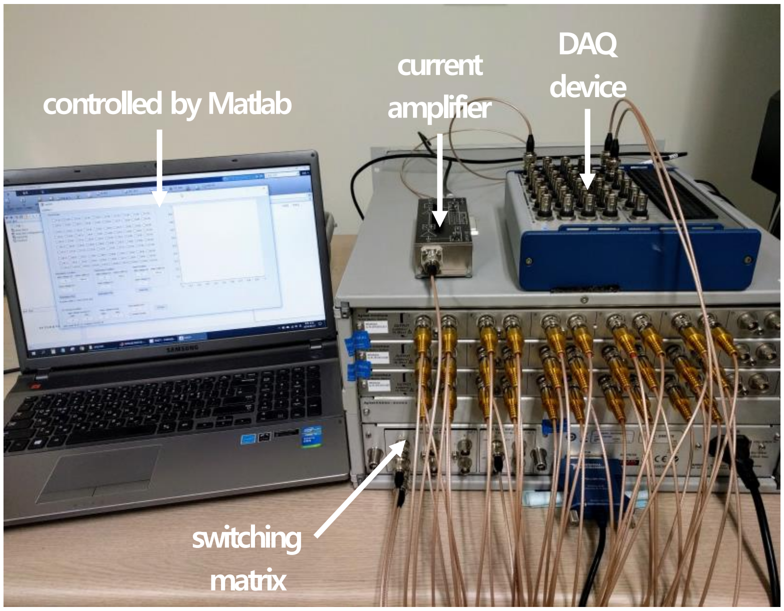

Figure S5. Photo of the measurement system which is connected to the test board.

Fig. S5 shows the photo of the measurement system which is connected to the test board. The control of all measurement equipment is carried out via home-made MATLAB program. When the application of a pulse signal to the test board is required, the pulse generated by DAQ equipment (NI USB-6363) is applied to the synaptic device array through the switching matrix (Agilent 5250A). When the amount of output current is to be measured, the output current is converted to the voltage by the current amplifier (FEMTO, DHPCA-100), and the converted voltage is measured through DAQ equipment.

Note 3. The comparison of the classification accuracy with/without negative synaptic weight value.

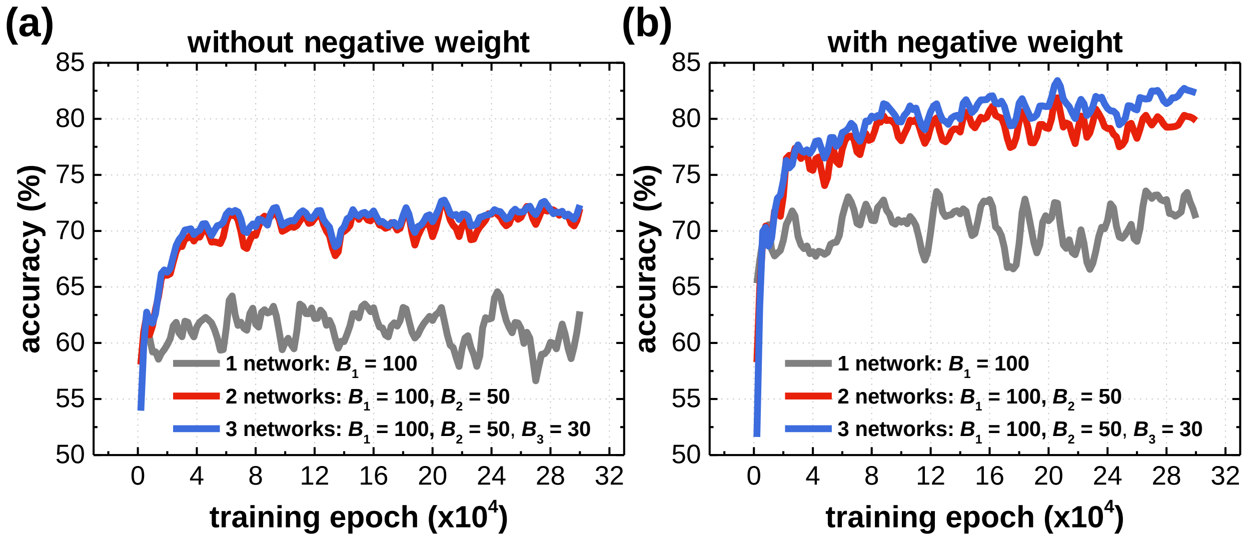

Figure S6. Classification accuracy as a function of training epoch (a) without negative synaptic weight, (b) with negative synaptic weight (it is the same as Fig. 3c), respectively.

As discussed in the main text, the one network (*e.g.*, G_1) is divided into two sub-networks; one of which represents a positive synaptic weight value ($G_{1\text{-}1}$) and the other of which represents a negative weight value ($G_{1\text{-}2}$). In fact, the network without $G_{1\text{-}2}$ can classify the patterns as shown in Fig. S6a, however, the accuracy is lower than that of the network with $G_{1\text{-}2}$ as shown in Fig. S6b. Because the negative synaptic weight has an information about opposite features of each label, the net weight ($G_{1\text{-}1}$–$G_{1\text{-}2}$) can enlarge the degree of inconsistency between the input and the trained pattern, which can improve the classification accuracy. The one drawback of this approach is that it requires twice as many synaptic devices. Therefore, depending on the complexity of the pattern to be classified or the application, it is appropriate to decide whether to use the sub-network of negative weight.

Note 4. The classification accuracy with different parameters (learning rate, bucket size)

4-1. Accuracy with different learning rates

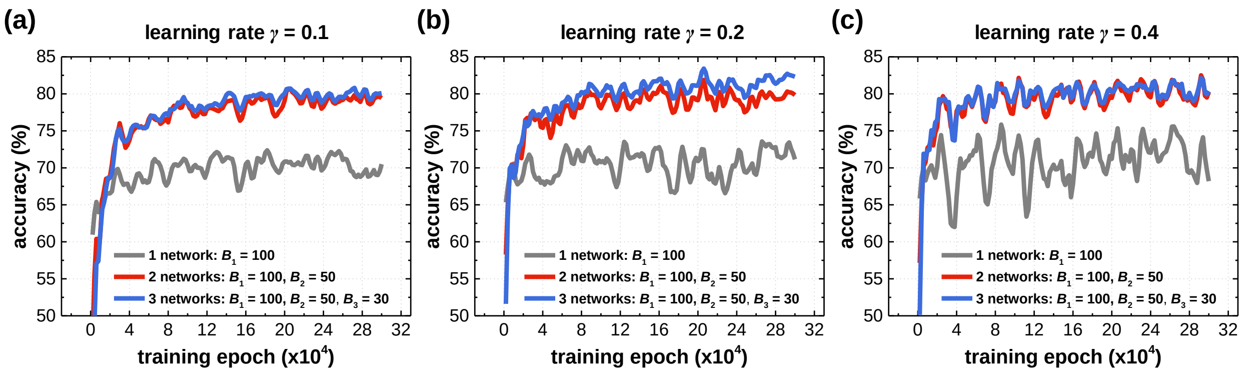

Figure S7. Classification accuracy according to different learning rate: (a) γ = 0.1, (b) γ = 0.2, and (c) γ = 0.4, respectively.

As discussed in the main text, write-vector $w(i)$ determines whether to adjust the synaptic weight according to the input patterns, which is stochastically determined as $w(i) = \{0, 1\}$ by the learning probability $P = \gamma \cdot u(i)$; here, γ is the learning rate. Fig. S7 shows the evolution of classification accuracy with different learning rate; as learning rate is increased, the evolution of accuracy is not stable and becomes very irregular. The reason of this instability is that more noise as well as input pattern information contributes to the write vector as the learning rate increases. Consequently, an unnecessary weight update due to the noise leads to a instability of the accuracy.

4-2. Accuracy with different bucket sizes

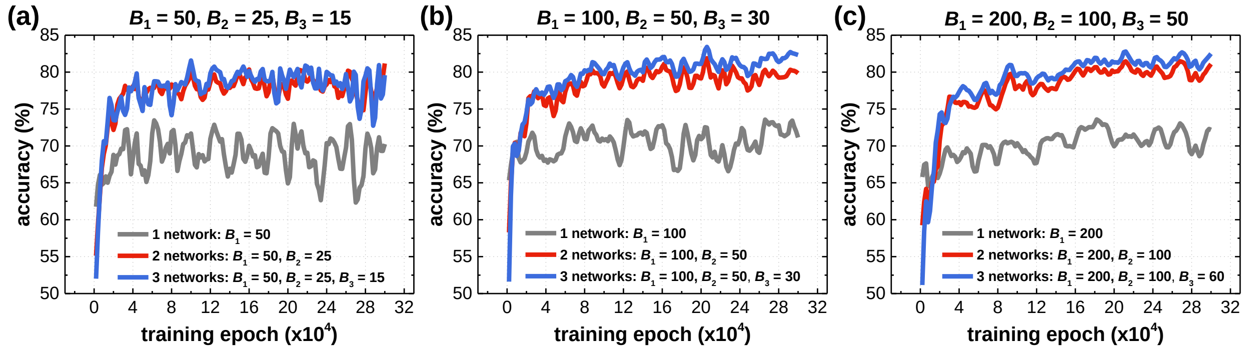

Figure S8. Classification accuracy according to different bucket sizes: a) B_1 = 50, B_2 = 25, B_3 = 15, b) B_1 = 100, B_2 = 50, B_3 = 30, and c) B_1 = 200, B_2 = 100, B_3 = 60, respectively. Learning rate γ is set to 0.2.

Fig. S8 shows the evolution of classification accuracy with bucket size. It is obvious that the evolution of accuracy is more stable and slightly improved accuracy can be obtained as the bucket size is increased. Since the number of synaptic weights corresponding to each input pattern label increases as the bucket size increases, the tolerance to the noise increases. Consequently, the evolution of accuracy becomes more stable, resulting in improved the accuracy.

Note 5. Face image classification

5-1. Yale face dataset

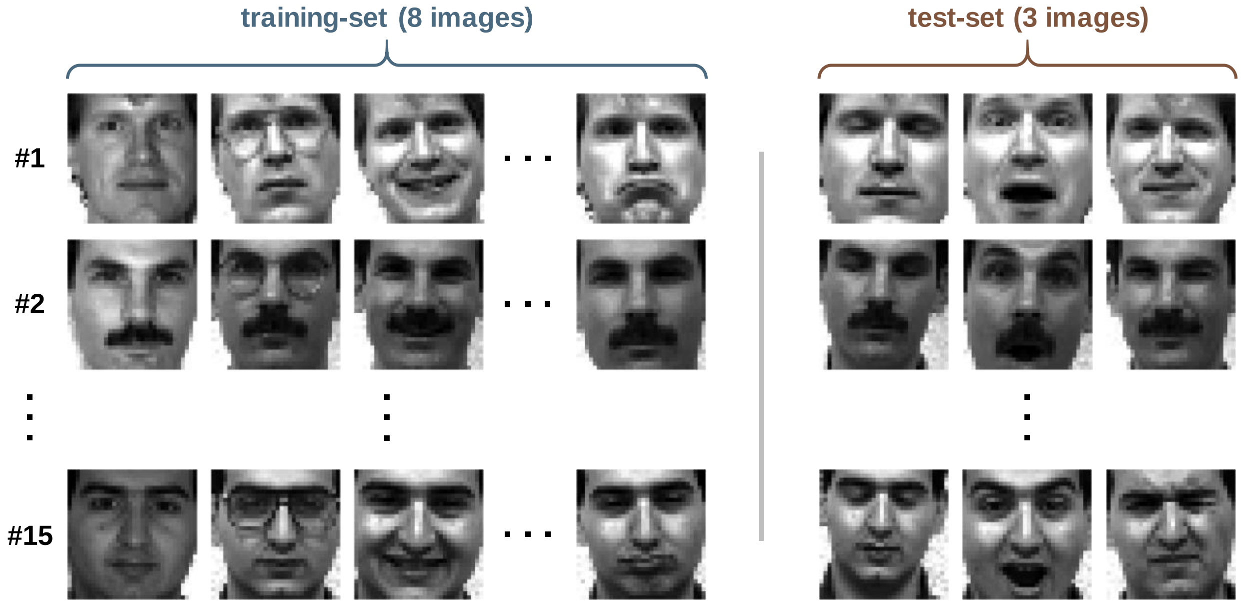

Figure S9. The example of Yale face dataset (32 × 32 pixels).

Fig. S9 shows the example of Yale face dataset. There are two version of the dataset; one with 32 × 32 pixels and the other with 64 × 64 pixels. The dataset contains 165 grayscale images of 15 individuals, thus there are 11 images per subject, one per different facial expression or configuration (center-light, with glasses, happy, left-light, without glasses, normal, right-light, sad, sleepy, surprised, and wink).

In our study, 8 of 11 images are used for training, and the remaining 3 images are used for testing. Therefore, only the images in the training-set are input to the network during the training phase. Then, to evaluate the classification accuracy, only the images in test-set are input during the recognizing phase.

5-2. The results of face image classification

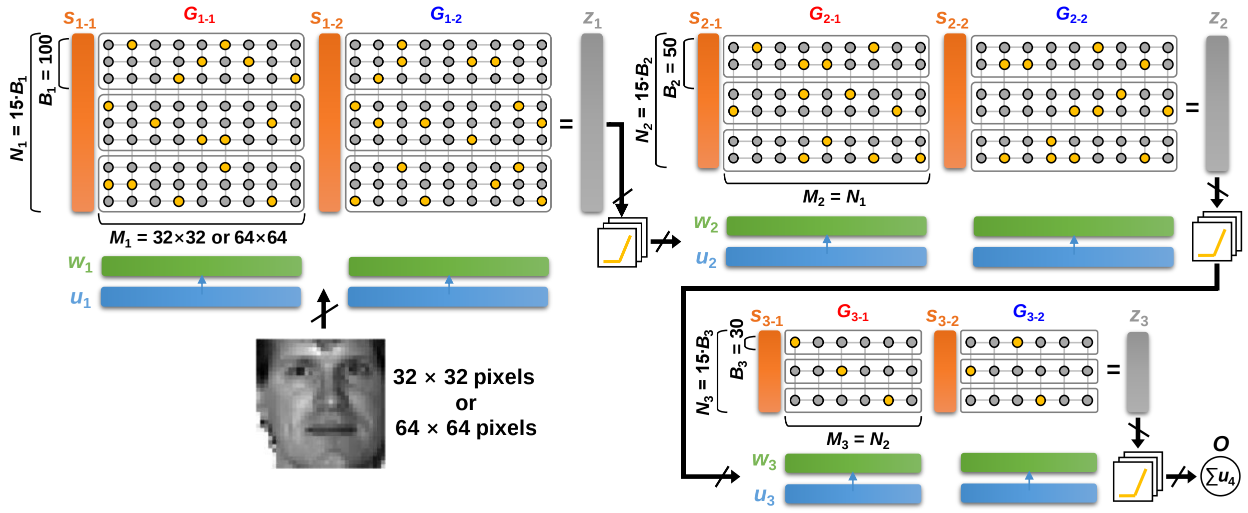

Figure S10. Schematic of the network architecture for face image classification with three networks (G_1, G_2, and G_3). The Bucket size of each network (B_1, B_2, and B_3) is fixed to 100, 50, and 30 respectively.

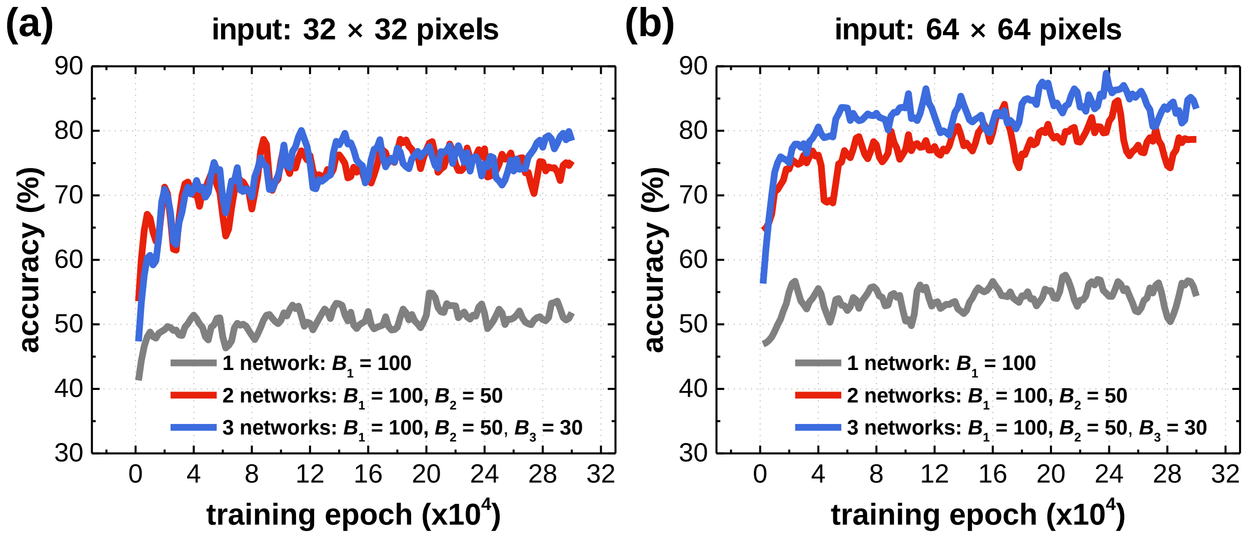

Figure S11. Classification accuracy of face images when the number of input image pixel is (a) 32 × 32, and (b) 64 × 64.

Fig. S10 shows the schematic of BNN including three networks (G_1, G_2, and G_3). The classification procedure is the same as that for MNIST dataset. The only difference is that the

number of pixels in the input pattern is increased from 28 × 28 (MINST) to 32 × 32 or 64 × 64 (Yale dataset), and the total number of labels in input pattern (*l*) is changed from 10 to 15.

The classification accuracy of face images is shown in Fig. S11 as a function of training epoch, where the number of networks alters the accuracy. With single network, the accuracy merely reaches approximately 50 % while deploying one more network improves the accuracy up to approximately 80 %. In addition, the training of clearer input pattern (*i.e.*, 64 × 64 pixels) leads to a higher classification accuracy. It is expected that the classification accuracy will be further improved through the optimization of network size or pre-process of input pattern. Consequently, total number of 599,700 synaptic transistors (599,700 bit = 73.2 Kbyte) are required to classify face images with 32 × 32 pixels, which results shows the potential of in-memory computing that can perform pattern classification without conventional logical calculation process.

Note 6. Additional experimental results of 3 × 3 binary pattern classifications

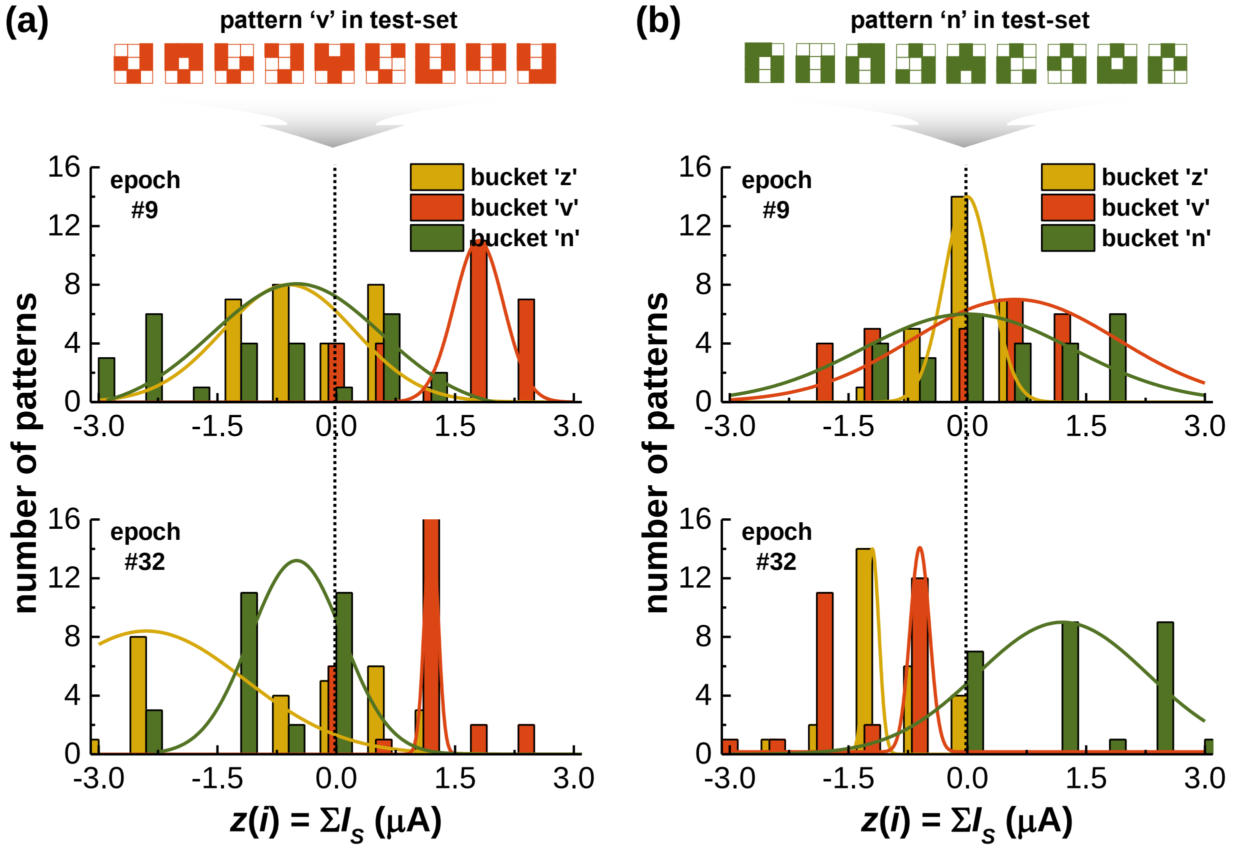

Figure S12. Obtained $z(i)$ (*i.e.*, integrated I_S in row direction) when training epoch is 9 and 32. (a) When the pattern 'v' is applied to the network during the recognizing phase, resultant $z(i)$ is different from each bucket; $z(i)$ obtained from bucket 'v' is much larger than the others. (b) Similar result can be obtained in the case of the pattern 'n'.

Figs. S12a and S12b show $z(i)$ in different training epoch. In the case of the pattern 'v' (Fig. S12a), $z(i)$ obtained from each bucket are almost similar when training epoch is 9. In contrast, when the training epoch is 32, $z(i)$ obtained from bucket 'v' is much larger than the others, which indicates that the pattern 'v' is classified. Similar results can be obtained in the case of the pattern 'n', as shown in Fig. S12b. Consequently, the pattern 'z', 'v', and 'n' can be classified accurately by evaluating $z(i)$ from each bucket.
